# Effectiveness of Using Augmented Reality for Training in the Medical Professions: Meta-analysis

**DOI:** 10.2196/32715

**Published:** 2022-07-05

**Authors:** Yahia Baashar, Gamal Alkawsi, Wan Nooraishya Wan Ahmad, Hitham Alhussian, Ayed Alwadain, Luiz Fernando Capretz, Areej Babiker, Adnan Alghail

**Affiliations:** 1 Faculty of Computing and Informatics Universiti Malaysia Sabah Labuan Malaysia; 2 Institute of Sustainable Energy Universiti Tenaga Nasional Kajang Malaysia; 3 Department of Computer and Information Sciences Universiti Teknologi Petronas Seri Iskandar Malaysia; 4 Department of Computer Science King Saud University Riyadh Saudi Arabia; 5 Department of Electrical & Computer Engineering Western University Ontario, ON Canada; 6 Department of Computer Engineering Future University Khartoum Sudan; 7 Department of World Languages Greece Central School District New York, NY United States

**Keywords:** augmented reality, medical, training, virtual, meta-analysis

## Abstract

**Background:**

Augmented reality (AR) is an interactive technology that uses persuasive digital data and real-world surroundings to expand the user's reality, wherein objects are produced by various computer applications. It constitutes a novel advancement in medical care, education, and training.

**Objective:**

The aim of this work was to assess how effective AR is in training medical students when compared to other educational methods in terms of skills, knowledge, confidence, performance time, and satisfaction.

**Methods:**

We performed a meta-analysis on the effectiveness of AR in medical training that was constructed by using the Cochrane methodology. A web-based literature search was performed by using the Cochrane Library, Web of Science, PubMed, and Embase databases to find studies that recorded the effect of AR in medical training up to April 2021. The quality of the selected studies was assessed by following the Cochrane criteria for risk of bias evaluations.

**Results:**

In total, 13 studies with a total of 654 participants were included in the meta-analysis. The findings showed that using AR in training can improve participants' performance time (*I*^2^*=*99.9%; *P*<.001), confidence (*I*^2^*=*97.7%; *P=*.02), and satisfaction (*I*^2^*=*99.8%; *P=*.006) more than what occurs under control conditions. Further, AR did not have any effect on the participants’ knowledge (*I*^2^=99.4%; *P=*.90) and skills (*I^2^=*97.5%; *P=*.10). The meta-regression plot shows that there has been an increase in the number of articles discussing AR over the years and that there is no publication bias in the studies used for the meta-analysis.

**Conclusions:**

The findings of this work suggest that AR can effectively improve performance time, satisfaction, and confidence in medical training but is not very effective in areas such as knowledge and skill. Therefore, more AR technologies should be implemented in the field of medical training and education. However, to confirm these findings, more meticulous research with more participants is needed.

## Introduction

Augmented reality (AR) is an interactive technology that uses persuasive digital data and real-world surroundings to expand the user's reality, wherein objects are produced by various computer applications [1,2]. Its use has become a widely debated topic among the medical community and has resulted in fertile ground for new types of research [3]. As a result, the health care sector and policy makers have been immediately able to realize the benefits of applying AR technologies, with education and training being noticeable applications of AR in medical settings [4-6]. However, since AR technologies enable learners to interact and visualize in 3D representations, medical professionals must have a vast amount of technological knowledge to efficiently adapt AR in practice [4,7,8]. Apart from the enormous benefits of AR for medical professionals, AR is also emerging as a useful guide for patient education and training, allowing physicians to clarify different surgical procedures and the functions of certain medications for patients and to explain new therapies in a more visual way [9-12]. Furthermore, AR can significantly improve a patient’s experience and advance knowledge on complex issues, and these benefits contribute and lead to better health outcomes [6].

There are many applications of AR in medical settings. Kamphuis et al [13], for example, reported that AR has reached a mature level of use in the field of anatomical engineering, for various technologies, and in physiological training. Further, surgical training has also been revolutionized by AR. Although integrating virtual reality (VR) and AR training into medical school and residency programs would necessitate prototype testing and entirely new teaching methods, AR has the potential to completely transform medical and science training [14-16]. Early adopters of AR innovations in medical training have noted several benefits, including the ability to simulate real-life scenarios without risking real-world consequences. Despite these advancements, there is still a lack of published experimental and observational work on the effectiveness and usefulness of AR in medical training and education [16].

In their work, Eckert et al [17] provided an overview of the development of AR applications in health care from 2012 to 2017. According to their findings, despite an increasing number of publications on AR in medicine, there have been no significant clinical trials on AR effectiveness. However, display-related domains tended to receive more research attention than other AR interventions.

The literature shows some discrepancies in the effectiveness of AR in medical training. To date, most of the meta-analysis research on the efficiency of VR and virtual patients in medical education has been carried out by Chen et al [18] and Kononowicz et al [19]. Their research has inspired this study, and there is some thematic overlap, but they presented their findings in a different way. However, to the best of our knowledge, no meta-analysis has been conducted to assess AR in medical training, with the exception of 2 studies—those by Williams et al [20] and Barsom et al [16], which conducted a systematic review with no meta-analysis. Therefore, filling this gap represents a significant addition to the literature. The primary goal of this study is to perform a meta-analysis on the efficacy of AR in 5 areas of learning and training. These include participants’ skills (eg, the ability to illustrate a certain process), knowledge (eg, the ability to understand a certain concept), confidence (eg, the ability to learn certain content with self-confidence), performance time (eg, the amount of time spent on a certain task), and satisfaction (eg, the level of learning fulfilment for a certain AR intervention). The findings of this work have many implications for policy makers, patients, medical students, and professionals and for using AR technology to facilitate learning and training mechanisms in the medical field. This meta-analysis seeks to address the following research questions (RQs):

- RQ1: What are the characteristics of studies using AR in medical training?
- RQ2: What kind of AR interventions were used to assist with medical training?
- RQ3: Does the use AR have an effect on medical training when compared to other methods?

## Methods

### Overview

The *Methods* section discusses the approaches used to obtain specific publications as well as the constraints and eligibility requirements that were used [21]. Following that, an overview of the standards used to interpret the studies and associated variables is provided. This meta-analysis was carried out in compliance with the PRISMA (Preferred Reporting Items for Systematic Reviews and Meta-Analyses) guidelines [22]. A checklist of all PRISMA items is presented in Multimedia Appendix 1.

### Eligibility Criteria

The meta-analysis included studies that (1) were published between 2013 and April 2021, (2) were written in English, and (3) primarily focused on AR for learning and training. Further, we excluded studies that (1) used other virtual platforms, such as VR and virtual patients, and (2) had no experimental data. However, based on the PICO (Population, Intervention, Comparison, Outcomes) framework, our final analysis incorporated various trials, including those with randomized controlled and mixed designs. Textbox 1 illustrates all of the PICO elements used for the inclusion criteria.

**PICO (Population, Intervention, Comparison, Outcomes) framework.**

**Criteria and descriptions**

- Population
  Sex (male or female)
  Age (>15 years)
  Target group (medical professionals, residents, patients, or students)
- Intervention
  All types of augmented reality technology used in training or learning.
- Comparison
  Augmented reality, nonaugmented reality, and conventional methods (eg, paper or pen, classes, simulations, or presentations)
- Outcomes
  Knowledge
  Confidence
  Skills
  Performance time
  Satisfaction

### Information Source and Search Strategy

We conducted a web-based literature search in the Cochrane Library, PubMed, Embase, Web of Science and Google Scholar databases from February to April 2021. We also manually checked the reference lists of the eligible studies to identify and acquire additional relevant publications. We used the following set of keywords, terms, and logical operators: “augmented reality” OR “AR” OR “mixed reality” OR “patient simulation” AND “medical training” OR “medical education” OR “health professions training.” Some of the search strategies and terms used are presented in Multimedia Appendix 2. It is worth mentioning that the search strategies were slightly modified to suit each web-based database. EndNote X9 (Clarivate) software was used to manage and import the selected documents and to remove duplicates. This process was carried out by 2 authors (YB and GA) of this study.

### Data Extraction

Data were placed into an extraction spreadsheet by using Microsoft Excel 2019. From each of the studies, information was extracted based on the study characteristics, designs, and participants. These data included the names of authors, the years of the studies, the locations of the studies, sample sizes, participant types, study designs, and interventions and outcomes. EndNote X9 was also used to obtain some publication data, such as titles, publishers, URLs, digital object identifiers, page numbers, issue numbers, and volume numbers. The process of data extraction was initially performed by 2 authors (HA and A Alwadain) and was then validated by another 2 authors (WNWA and LFC).

### Risk of Bias Assessment

The quality of the studies was assessed by 2 authors (AB and A Alghail). We followed the criteria of the Cochrane Handbook for Systematic Reviews of Interventions [23] in the risk of bias assessment process. These include 7 domains that correspond to a particular kind of bias. Each domain was given a score (a “high risk,” “low risk,” or “unclear risk” of bias), and any disagreements among coauthors were settled via consensus.

### Statistical Analysis

RevMan 5.4 (The Cochrane Collaboration) software was used to perform the risk of bias assessment; different scales were used by different authors to measure the outcomes; therefore, raw data could not be compared directly. Thus, the R 4.0.2 (R Foundation for Statistical Computing) software was used to transform raw data, so that STATA 14.0 (StataCorp LLC) could be used to run the analysis directly. The *metacont* package on R was used to transform the data on the sample sizes, SDs, and means of the control and AR groups. Data on the sample sizes, means, and SDs of only the AR groups were transformed by using the *metamean* package. Only 1 study [24] recorded the sample size and the proportion of participants on whom AR had a positive effect. The effect sizes and CIs were generated by using the two different packages. STATA 14.0 was used to generate the forest plot for each outcome, which used the transformed data (effect size and CI), via the *metan* command. The statistical heterogeneity among the selected studies was measured by using *I*^2^ in each analysis and a 5% significance level. The random effect model was selected for the analysis carried out because the true effect sizes underlying all studies were stochastic.

## Results

### Study Selection

**Figure 1:**
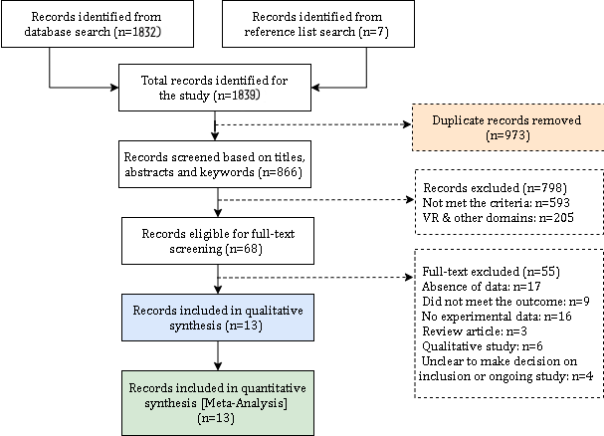
Study screening and selection flowchart. VR: virtual reality.

From our initial search of 5 potential databases, we found a total of 1832 records. We identified a total of 1839 records, including relevant reference lists (n=7). Duplicate records (973/1839, 52.9%) were removed. Following that, a total of 866 of the 1839 (47.1%) studies were screened based on their titles, abstracts, and keywords, and 798 were excluded after applying the eligibility criteria. A total of 68 studies were downloaded and evaluated via full-text screening, of which 55 were excluded for various reasons (Figure 1). Finally, 13 studies with a total of 654 participants were eligible for the meta-analysis; 2 studies used the same set of participants for both the control and AR groups [25,26], while another 6 studies [27-32] that also measured both the control and AR groups divided the sample size.

### Study Characteristics

As presented in Table 1, the trials of the selected studies were performed in the following ten countries: Germany [27,28,30], the United States [24,31], the United Kingdom [33], Canada [34], Italy [35], Sweden [26], Finland [32], Switzerland [29], Spain [36], and South Korea [25]. Furthermore, 10 of the 13 studies, adopted a randomized control trial [24,26-29,32-36], 2 studies had a mixed design [30,31], and 1 study used a cohort approach [25]. The number of participants in the studies ranged between 4 and 372. With regard to these participants, 5 trials recruited medical and nursing students [25,27,28,30,31]; 2 trials involved pediatric and psychiatry residents [26,29]; 1 trial recruited visitors to a center [32]; and the remaining trials (n=5) included multiple participants, such as physicians, registered nurses, technicians, residents, students, clinicians, clients, hosts, and paramedics [24,33-36].

In recent years, the use of AR in the medical field has attracted the attention of academics and researchers, as evidenced by the increasing number of publications devoted to AR. This is demonstrated by the fact that 8 of the 13 studies were conducted between 2019 and 2021. The characteristics of the selected studies, including the outcome measures, interventions, and types of participants, are shown in Table 1. Additional details regarding the AR and control groups are presented in Multimedia Appendix 3.

**Table 1:** Characteristics of the selected studies.

Author, year	Country	Brief description	Participants	Sample size, n (number of participants in each study group)	Study design	Outcomes
Albrecht et al, 2013 [27]	Germany	Comparing the effect of MAR^a^ to that of a textbook, with consideration for essential psychological qualities	Students	10 (6 and 4)	RCT^b^	Skill and confidence
Balian et al, 2019 [24]	United States of America	Testing the feasibility of an AR^c^ training system (Microsoft HoloLens) for CPR^d^ among medical professionals	Physicians, nurses, and technicians	51 (N/A^e^)	RCT	Performance time and satisfaction
Ingrassia et al, 2020 [35]	Italy	Assessing the feasibility of an AR prototype for BLSD^f^ training	Physicians, nurses, and residents	26 (N/A)	RCT	Confidence and satisfaction
Kim et al, 2021 [25]	Korea	Evaluating the usability and feasibility of AR (smart glasses) for nursing training skills	Students	30 (N/A)	Cohort	Skill, performance time, satisfaction, and knowledge
Kotcherlakota et al, 2020 [31]	United States of America	Assessing the use of AR (clinical simulation) in the management of pediatric asthma outcomes	Students	21 (12 and 9)	Mixed	Confidence and satisfaction
Muangpoon et al, 2020 [33]	United Kingdom	Proposing an AR system (Microsoft *HoloLens, 1st Gen*) to improve the learning and teaching of DRE^g^	Clinicians and students	19 (N/A)	RCT	Skill, knowledge, and satisfaction
Noll et al, 2017 [28]	Germany	Assessing learning success by comparing learners with and without MAR	Students	44 (22 and 22)	RCT	Knowledge and skill
Pantziaras et al, 2015 [26]	Sweden	Evaluating the impact of virtual patient training on the knowledge of stress disorder management and symptoms	Residents	32 (N/A)	RCT	Knowledge
Savela et al, 2020 [32]	Finland	Investigating the features of MAR for learning and sociability	Visitors	372 (231, 71, and 71)	RCT	Knowledge and satisfaction
Schiffeler et al, 2019 [30]	Germany	Assessing the effects of AR on interaction and communication	Students	13 (7 and 6)	Mixed	Knowledge
Siebert et al, 2017 [29]	Switzerland	Evaluating whether the adaption of AR glasses with AHA^h^ guidelines can reduce the time and deviation of essential lifesaving exercises throughout pediatric CPR when compared to those of PALS^i^	Residents	20 (10 and 10)	RCT	Performance time and confidence
Vidal-Balea et al, 2021 [36]	Spain	Evaluating MAR games that teach and train people how to use pediatric medical applications (the games also monitor training progress)	Clients and hosts	4 (N/A)	RCT	Performance time
Wang et al, 2017 [34]	Canada	Developing a telemedicine platform using AR (Microsoft HoloLens) to improve medical training remotely	Paramedics and students	12 (N/A)	RCT	Performance time and satisfaction

^a^MAR: mobile augmented reality.

^b^RCT: randomized controlled trial.

^c^AR: augmented reality.

^d^CPR: cardiopulmonary resuscitation.

^e^N/A: not applicable.

^f^BLSD: basic life support and defibrillation.

^g^DRE: digital rectal examination.

^h^AHA: American Heart Association.

^i^PALS: pediatric advanced life support.

### Risk of Bias

**Figure 2:**
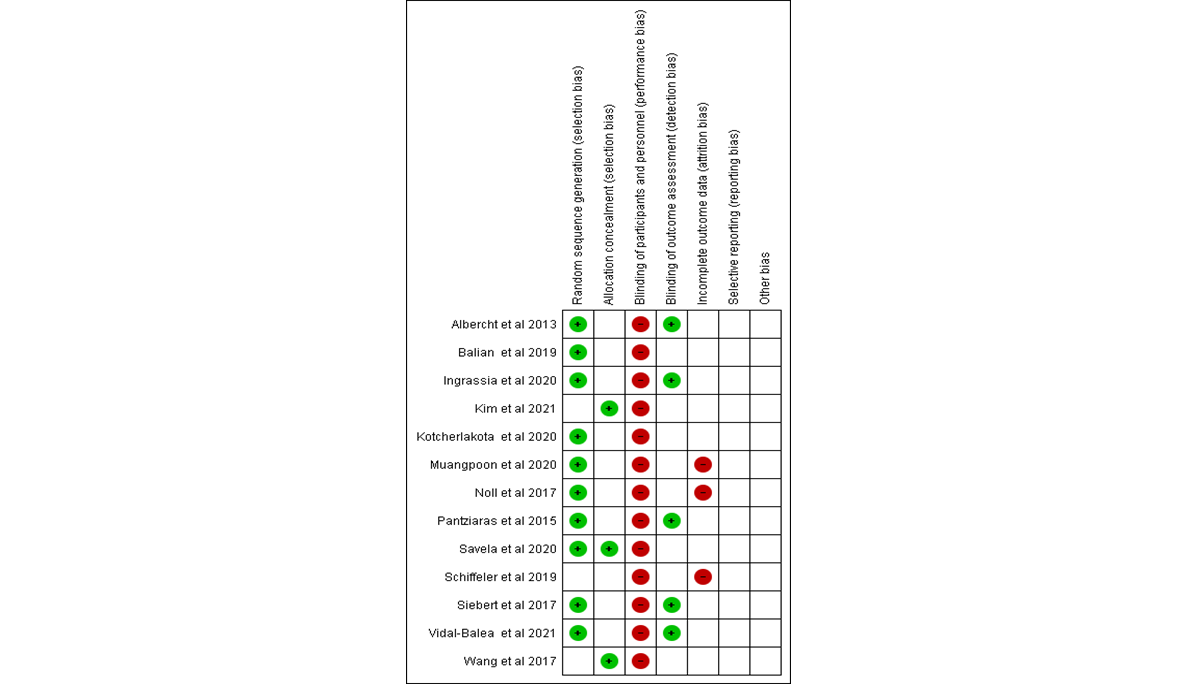
Risk of bias assessment of each selected study [24-36].

**Figure 3:**
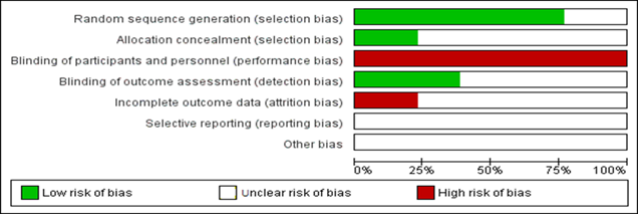
Overall risk of bias assessment of the selected studies.

Figure 2 and Figure 3 present the risk of bias assessment, which was performed according to the Cochrane criteria. Of the 13 studies, 10 detailed randomized methods [24,26-28,30-33,35,36], while the rest of the studies (n=3) [25,29,34] reported no sequence generation methods. Only 3 trials reported concealment methods [25,32,34]. Furthermore, 3 studies reported dropouts [29,30,33] but did not go into detail about how they were handled. Due to the uniqueness of the intervention methods, no blinding methods were reported for participants in any of the trials. The blinding of assessors was used in 5 trials [26,27,29,35,36].

### Publication Bias

**Figure 4:**
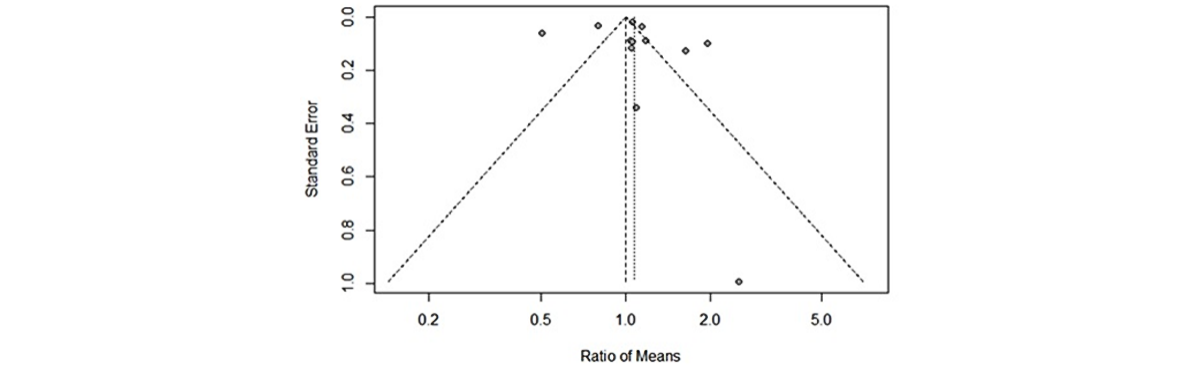
Funnel plot showing publication bias.

The most significant issue with meta-analyses is the possibility of bias and error arising from combining multiple studies, especially unrelated studies. Creating a funnel plot is one of the key methods used for testing common publication bias. The graphics in the funnel are obvious scatter plots for the effect size approximated in each selected study versus the unit of sample size reported in the studies [37]. If there is no bias, the graphic resembles an upside-down funnel. However, if a publication bias exists, the graphic should be asymmetric and distorted [38]. Figure 4 shows the funnel plot that we used to test for publication bias.

As can be seen in Figure 4, the 13 studies included in the meta-analysis are symmetrically placed on both sides of the vertical effect size line and are very close to the symmetrical and merged effect size lines. If there is no publication bias, studies should be distributed symmetrically on both sides of the vertical line depicting the merged effect size. If the studies are placed outside of the pyramid, they should be concentrated in the upper or middle parts of the plot. However, if there is a bias, most of the studies will be in the funnel plot's bottom part or in only 1 vertical line segment [39]. The effect sizes of the included studies are distributed in a symmetrical manner, with only 5 studies located outside of the pyramid. However, it is worth nothing that 4 of these studies are located in the upper part of the pyramid. This pattern shows that the studies included in this work have no publication bias.

**Figure 5:**
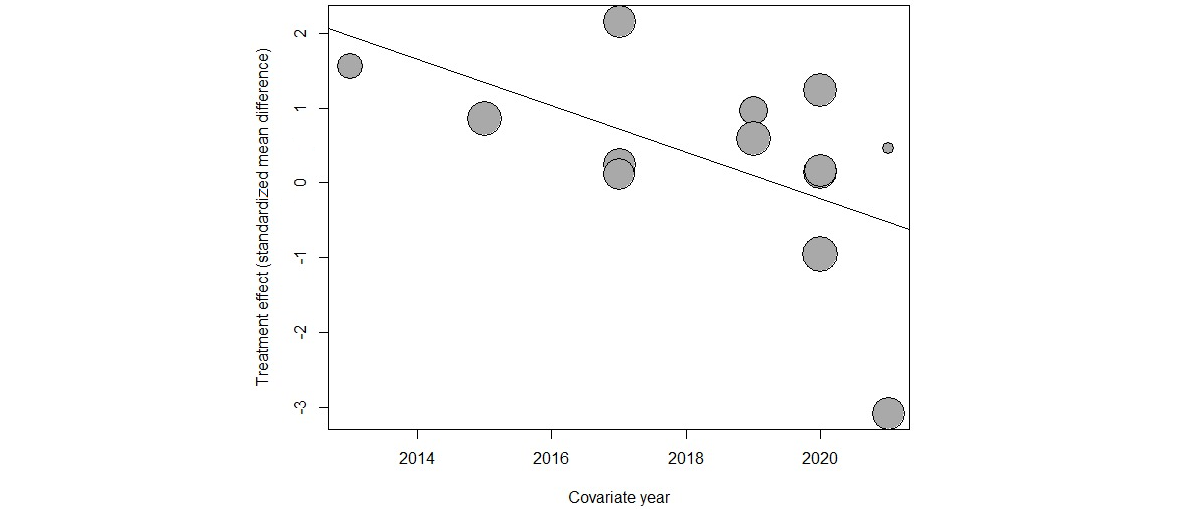
Meta-regression plot showing the publication years.

As shown in Figure 5—the meta-regression plot—the true effect sizes between the control and AR groups are 1.8 for the 2013 study [27]; 0.9 for the 2015 study [26]; 2.2, 0.2, and −0.9 for the 2017 studies [28,29,34], respectively; 1.2 and 0.9 for the 2019 studies [24,30], respectively; 0.4, −1, 0.2, and 1.5 for the 2020 studies [31-33,35], respectively; and finally, −3.1 and 0.8 for the 2021 studies [25,36], respectively.

### Results of the Meta-analysis

A meta-analysis was performed by using the data set in Multimedia Appendix 4 for each of the five outcomes (ie, knowledge [Multimedia Appendix 5], performance time [Multimedia Appendix 6], satisfaction [Multimedia Appendix 7], confidence [Multimedia Appendix 8], and skills [Multimedia Appendix 9]). The coding of the final analysis is also provided in Multimedia Appendix 10.

#### Knowledge

**Figure 6:**
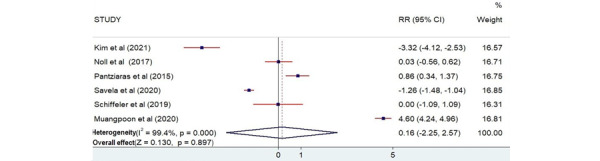
Forest plot showing the effectiveness of augmented reality on knowledge [25,26,28,30,32,33]. Weights are from the random-effects model. RR: risk ratio.

Only 6 of the 13 selected studies recorded knowledge as scores [25,26,28,30,32,33]. The total number of participants who measured their knowledge was 510. The findings from the plot (Figure 6) showed that there is no relationship between AR training and improvement in the participants' knowledge (*Z*=0.130; *P*=.90).

#### Skills

**Figure 7:**
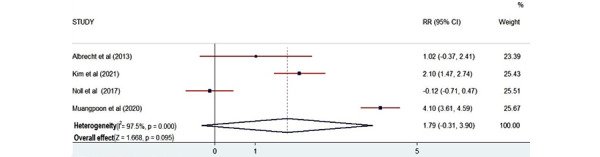
Forest plot showing the effectiveness of augmented reality on skills [25,27,28,33]. Weights are from the random-effects model. RR: risk ratio.

A total of 4 studies used skill as the outcome measurement [25,27,28,33]. The total number of participants that participated in measuring skill was 103. The plot (Figure 7) revealed that there is no statistical relationship between AR training and participants’ skills (*Z*=1.668; *P*=.10). There is high heterogeneity among the studies.

#### Confidence

**Figure 8:**
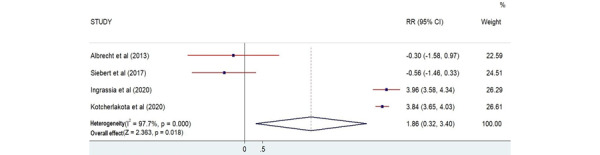
Forest plot showing the effectiveness of augmented reality on confidence [27,29,31,35]. Weights are from the random-effects model. RR: risk ratio.

In total, 4 studies recorded confidence outcomes [27,29,31,35]. The total number of participants who measured their confidence was 77. There was a statistical relationship between AR training and participants’ confidence, as the *P* value was significant according to the plot (*Z*=2.363; *P*=.02; Figure 8).

#### Performance Time

**Figure 9:**
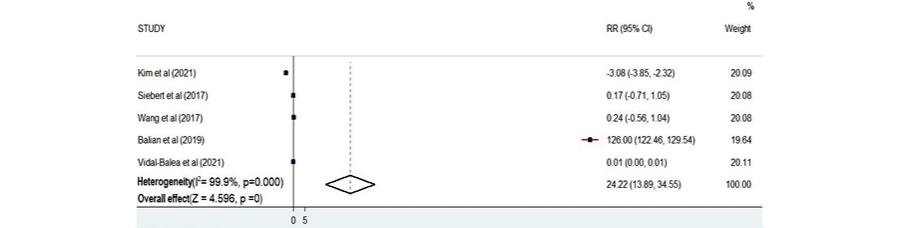
Forest plot showing the effectiveness of augmented reality on performance time [24,25,29,34,36]. Weights are from the random-effects model. RR: risk ratio.

Performance time was assessed as an outcome measure in 5 studies [24,25,29,34,36]. The total number of participants that participated in measuring performance time was 129. There was a statistical relationship between AR training and participants’ performance time because the *P* value was significant according to the plot (*Z*=4.596; *P*<.001; Figure 9).

#### Satisfaction

**Figure 10:**
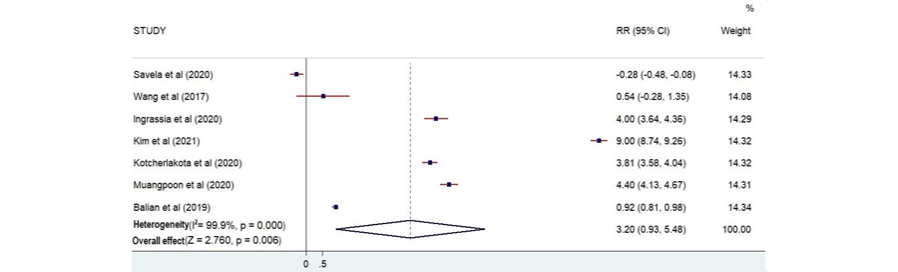
Forest plot showing the effectiveness of augmented reality on satisfaction [24,25,31-35]. Weights are from the random-effects model. RR: risk ratio.

A total of 7 studies reported participants’ satisfaction [24,25,31-35]. The total number of participants who measured their satisfaction was 543. There was a statistical relationship between AR training and participants’ satisfaction according to the plot (*Z*=2.760; *P*=.006; Figure 10).

## Discussion

### Main Findings

The usefulness of AR simulation methods in medical training was evaluated in this meta-analysis. In terms of skill outcomes, AR training did not outperform other educational methods for medical training. A study on the use of AR in medical education found that students’ skills improved after the intervention [25]. In terms of skill performance scores and success rates, it was found that AR groups did not exhibit any statistical relationships, even though each of the trials that reported skills (ie, those included in our study) used different education and training methods for their control groups.

There was a statistical difference (*P*=.02) between the AR and control groups in terms of confidence. Participants' confidence was improved by AR when compared to that of participants in control conditions. It was found that AR training had a greater effect on participants’ confidence.

The *I*^2^ values reported throughout the *Results* section are very high, indicating that there was a huge level of heterogeneity between studies. In terms of knowledge outcomes, participants who underwent AR training did not outperform those who used other educational methods for medical training. However, a previous study that looked at how AR affects learning found that AR was more effective for knowledge retention [27] and that students are more likely to make connections between concepts when they are in an interactive learning environment. As a result, more research into the impact of AR on knowledge in medical training is needed in the future.

Between the control and AR groups, there was a significant difference in participants’ satisfaction (*P*=.006). Of the 7 studies that recorded satisfaction as an outcome, 4 [25,32-34] indicated partial satisfaction. Some participants remarked on how difficult it is for AR technology to affect user satisfaction. As a result, we believe that participants’ satisfaction with AR training may vary depending on technical factors. AR technology will be better able to satisfy users as the technology advances.

A meta-analysis of performance time was also carried out. The findings suggested that AR was more effective than other training methods at reducing performance time. However, we found that AR was more efficient than other methods in enhancing performance time. The observed heterogeneity could have been due to the various research designs and settings used in the selected studies, such as surgical projects, AR devices, and control group training methods. One study [36] on the efficiency of AR endoscopy simulation training analyzed performance time based on real-world data and found no significant difference between the AR and control groups, but the evidence quality was poor. Other studies that measured performance time as an outcome found that AR can assist operators in reducing performance time [24,25].

### Strengths and Limitations

One of the main strengths of this work is being among the first meta-analyses to be conducted on the effectiveness of AR in medical training, which presents a significant contribution to the advancement of AR in the medical field. Furthermore, our work involved an assessment of 13 trials with a total of 654 participants from various countries.

Our work has some limitations as well. The first is that we only included articles that focused on AR as an intervention, which made it difficult to find a large number of studies. Second, some of the studies included in the review omitted information about allocation concealment, sequence generation, and blinding methods. Finally, the 13 trials we evaluated used different teaching and training methods for their control groups, which could lead to significant heterogeneity.

### Conclusions

The effectiveness of AR training methods in the medical field was assessed in this work. The meta-analysis included 13 trials with a total of 654 participants that were completed between 2013 and 2021. In all trials, AR training was used as an intervention in AR groups, while conventional methods were used for control groups. Based on our findings, medical students' performance time, confidence, and satisfaction can be improved by using AR training and education methods. However, there was no statistical difference between the skills and knowledge that participants gained by undergoing AR training and those gained via conventional training methods. The use of AR should therefore be adopted in medical department training because of its significant effect on the performance time of participants. In general, AR should be used to expand knowledge and as a supplement to other simulation approaches to enhance clinical practice quality and safety. It is very noticeable that there are considerable gaps in the literature regarding the use of AR technology in medical training, and a limited number of studies exist, suggesting that further research efforts in this area are needed.

## Appendix

Multimedia Appendix 1 PRISMA (Preferred Reporting Items for Systematic Reviews and Meta-Analyses) checklist.

Multimedia Appendix 2 Search strategies.

Multimedia Appendix 3 Augmented reality and control groups.

Multimedia Appendix 4 The data set.

Multimedia Appendix 5 Knowledge.

Multimedia Appendix 6 Performance time.

Multimedia Appendix 7 Satisfaction.

Multimedia Appendix 8 Confidence.

Multimedia Appendix 9 Skill.

Multimedia Appendix 10 R and Stata codes.

## Abbreviations

AR: augmented reality
PICO: Population, Intervention, Comparison, Outcomes
PRISMA: Preferred Reporting Items for Systematic Reviews and Meta-Analyses
RQ: research question
VR: virtual reality
