# Supplementary material for: Effectiveness of Using Augmented Reality for Training in the Medical Professions: Meta-analysis
Source: JMIR Serious Games. 2022 Jul 5;10(3):e32715. doi: 10.2196/32715 (PMC9297143; doi:10.2196/32715)
Supplement: Multimedia Appendix 2 [file games_v10i3e32715_app2.docx]

**Multimedia Appendix 2. Search strategies.**

**PubMed**

#1. Augmented Reality OR AR [Mesh]

#2. Patient Simulation OR Virtual Patient [Majr]

#3.((("Augmented reality"[Title/Abstract]) OR (''patient simulat*"[Title/Abstract])) OR ("virtual patient*"[Title/Abstract])) #4. #1 OR #2 OR #3

#5. Medical Education OR Training [Mesh]

#6.(("Medical Education"[Title/Abstract]) OR ("Education of medical department"[Title/Abstract]) OR ("Training of medical department"[Title/Abstract]))

#7. #5 OR #6

#8. #4 AND #7

**Embase**

#1. ‘AR’ OR 'Augmented reality'/exp

#2. 'virtual reality’: ab,ti OR 'patient simulation*':ab,ti

#3. #1 OR #2

#4. 'Medical education'/exp

#5. 'medical* education':ab,ti OR 'training in medical department':ab,ti OR 'medical education & training':ab,ti

#6. #4 OR #5

#7. #3 AND #6

**The Cochrane Library**

#1. MeSH descriptor: [Augmented reality] this term only

#2. ("Augmented reality"):ti,ab,kw OR (''patient simulation*"):ti,ab,kw OR ("Augmented simulation"):ti,ab,kw

#3. #1 OR #2

#4. MeSH descriptor: [Education, medical] this term only

#5. ("medical* education"):ti,ab,kw OR ("education of medicine"):ti,ab,kw OR ("medical, training"):ti,ab,kw

#6. #4 OR #5

#7. #3 AND #6
