# Supplementary material for: Effectiveness of Using Augmented Reality for Training in the Medical Professions: Meta-analysis
Source: JMIR Serious Games. 2022 Jul 5;10(3):e32715. doi: 10.2196/32715 (PMC9297143; doi:10.2196/32715)
Supplement: Multimedia Appendix 3 [file games_v10i3e32715_app3.doc]

**Multimedia Appendix 3. Augmented reality and control groups.**

| **Author/year** | **AR group** | **Control group** |
| --- | --- | --- |
| **Wang et al (2017)** | (A) The study looked at how the Microsoft HoloLens can be used to improve and facilitate remote medical training. AR systems have inherent advantages that allow remote learners to perform complex medical procedures such as Point of Care. Without visual interference, ultrasound (PoCUS). The HoloLens is used in this study to capture a first-person view of a simulated rural emergency room (ER) using mixed reality capture (MRC), and it also serves as a novel telemedicine platform with remote pointing capabilities. The hand of the mentor  Gestures are captured with a Leap Motion and virtualized in the HoloLens' AR space. | (B)Traditional education: written instructions. |
| **Muangpoon et al (2020)** | (A) The system was created using the Unity game engine (Unity Technologies) and an augmented reality device called a first-generation HoloLens (Microsoft Inc). The HoloLens is fully immersive and see-through, allowing the user to experience the environment as realistically as possible. Interactions with holographic content are also possible. The HoloLens has an inertial measurement unit (accelerometer, gyroscope, and magnetometer), four environment understanding sensors (two on each side), a 120° 120° angle of view energy-efficient depth camera, a 2.4-megapixel photographic video camera, a four-microphone array, and an ambient light sensor. | (B)Non-VR simulation: F2F high-fidelity manikin clinical simulation. |
| **Kim et al (2021)** | (A) Augmented reality techniques (Vuzix Blade) which is similar to that of ordinary glasses, and it supports voice recognition and touchpads. | (B)Non-AR simulation: XR image guide training program |
| **Vidal-Balea et al (2021)** | (A) Mobile AR Application interface and interactions with the users have been devised to be as simple as possible, with visual cues and minimizing the amount of text to be read. | (B)Non-AR simulation: Mannequin-based simulation, using live, professional facilitated, mannequin-based simulation. |
| **Savela et al (2020)** | (A) AR app intravenous simulator system that uses a haptic device, which requires physical contact between computer and user, a software program, a desktop or laptop computer. | (B) Traditional education: regularly scheduled learning activities.  Injection training, containing visible, palpable venous vessels. |
| **Albrecht et al (2013)** | (A) mARble is an iOS application that was developed at the Peter L. Reichertz Institute for Medical Informatics (PLRI) at the Hannover Medical School, mARble was able to detect and interpret predefined markers representing various pathologies commonly found in forensic medicine. Each marker corresponded to a wound pattern that the students were expected to explore. | (B) Traditional education: written instructions. |
| **Siebert et al (2017)** | (A) AR glasses (Google Glass, allocation group A) or AHA PALS conventional pocket reference cards (allocation group B) during a standardized simulation-based pediatric CA scenario. | (B)Non-AR simulation: Mannequin-based simulation led by a trained simulation facilitator. |
| **Noll et al (2017)** | (A) The iPhone operating system (iOS, Apple Inc)-based app mARble-Derma (mARble-dermatology, It provides users with learning content organized in the form of digital flashcards. Using paper-based markers that can be placed on the skin of users, the app employs AR to recall content linked to the markers, overlay it on images of the environment if desired, and to thus add an entirely new level of information | (B)Non-VR simulation: Mannequin-based simulation led by a trained simulation facilitator. |
| **Ingrassia et al (2020)** | (A) Holo-BLSD was developed jointly by the SIMNOVA simulation center (Novara, Italy) and the Department of Computer Engineering of Politecnico di Torino, in collaboration with Logosnet’s e-REAL Immersive Simulation Labs in Lugano, Switzerland. The app uses Microsoft’s HoloLens device, a wearable headset for AR experiences, and meets the recent American Heart Association guidelines | (B)Non-AR simulation: Low-fidelity simulator and realistic environment, guided throughout by the regular subject teacher. |
| **Pantziaras et al (2015)** | (A) AR system called Refugee Trauma Simulation (RT-SIM) that portrays an adult Bosnian refugee. An individualized, automated feedback module provided by both the VP and a virtual advisor (VA) follows. The feedback by the VP gives the patient’s perspective of the consultation, while the feedback by the VA focuses on more technical and clinical aspects of PTSD diagnostic criteria, clinical management, and basic communication skills | (B)Traditional education: written instructions. |
| **Balian et al (2019)** | (A) CPR training manikin was integrated with a commercial AR device (Microsoft HoloLens) to provide participants with real-time audio-visual feedback via a holographic overlay of blood flow to vital organs dependent on CC quality. In this system, higher quality CC visually improved virtual blood circulation. HCPs performed a 2-minute cycle of hands-only CPR using only the AR system, and CC parameters were recorded. | (B)Traditional education: practice session, supervised by an experienced faculty member and supplies were provided to practice CPR. |
| **Kotcherlakota et al (2020)**  **Schiffeler et al (2019)** | (A) Students used the AR ARIS app on their iPad to prioritize what to do first, as though in a patient care setting. This process allowed for making connections between classroom learning and clinical practice  A) The user interface and interactions with the Mobile AR Application have been designed to be as simple as possible, with visual cues and minimal text to read. | (B)Traditional education: regularly scheduled learning activities.  B) Traditional education: written instructions. |
