# Supplementary material for: Effectiveness of Using Augmented Reality for Training in the Medical Professions: Meta-analysis
Source: JMIR Serious Games. 2022 Jul 5;10(3):e32715. doi: 10.2196/32715 (PMC9297143; doi:10.2196/32715)
Supplement: Multimedia Appendix 10 [file games_v10i3e32715_app10.docx]

**Multimedia Appendix 10. R and Stata codes.**

***R Code:***

library(meta)

mydata<-read.csv(file.choose(),header=T)

mydata

str(mydata)

meta1<-metacont(n2,mean2,sd2,n1,mean1,sd1,data=mydata,studlab=paste(study),fixed = FALSE,random = TRUE,sm = "SMD")

forest(meta1,col.diamond="black",col.diamond.lines="red",squaresize = 0.5, col.square="blue",col.square.lines="black",just="center",xlab="Effect Size",smlab ="CFL MODERATE",xlim = "symmetric",test.overall = TRUE,prediction = TRUE, ff.smlab = "italic",ff.axis="bold")

mydata<-read.csv(file.choose(),header=T)

str(mydata)

meta2<-metaprop(n,N,data=mydata,studlab=paste(study),comb.fixed = FALSE,comb.random = TRUE,sm = "PRAW")

forest(meta2,col.diamond="black",col.diamond.lines="red",squaresize = 0.5, col.square="blue",col.square.lines="black",just="center",xlab="Effect Size",smlab ="CFL MODERATE",xlim = "symmetric",test.overall = TRUE,prediction = TRUE, ff.smlab = "italic",ff.axis="bold")

mydata<-read.csv(file.choose(),header=T)

str(mydata)

meta3<-metamean(n,mean,sd,data=mydata,studlab=paste(study),comb.fixed = FALSE,comb.random = TRUE,sm = "MRAW")

forest(meta3,col.diamond="black",col.diamond.lines="red",squaresize = 0.5, col.square="blue",col.square.lines="black",just="center",xlab="Effect Size",smlab ="CFL MODERATE",xlim = "symmetric",test.overall = TRUE,prediction = TRUE, ff.smlab = "italic",ff.axis="bold")

***Stata Code:***

ssc install metan

metan es lci uci

metan  es lci uci, lcols(study) xlabel(0,10) xsize(18) ysize(10) random effect(RR) ciopt(lcolor(red)) boxopt(mcolor(blue))
